# A Comparative Study between Olanzapine and Risperidone in the Management of Schizophrenia

**DOI:** 10.1155/2014/307202

**Published:** 2014-08-26

**Authors:** Saeed Shoja Shafti, Mahsa Gilanipoor

**Affiliations:** ^1^University of Social Welfare and Rehabilitation Sciences (USWR), Razi Psychiatric Hospital, P.O. Box 18735-569, Tehran, Iran; ^2^Razi Psychiatric Hospital, P.O. Box 18735-569, Tehran, Iran

## Abstract

*Introduction*. Since a variety of comparisons between risperidone and olanzapine have resulted in diverse outcomes, so safety and efficacy of them were compared again in a new trial. *Method*. Sixty female schizophrenic patients entered into one of the assigned groups for random allocation to olanzapine or risperidone (*n* = 30 in each group) in a double-blind, 12-week clinical trial. Scale for Assessment of Positive Symptoms (SAPS) and Scale for Assessment of Negative Symptoms (SANS) were used as the primary outcome measures. Clinical Global Impressions-Severity Scale (CGI-S), Schedule for Assessment of Insight (SAI), and finally Simpson Angus Scale (SAS) as well were employed as secondary scales.* Results*. While both of olanzapine and risperidone were significantly effective for improvement of positive symptoms (*P* < 0.0001), as regards negative symptoms, it was so only by means of olanzapine (*P* < 0.0003). CGI-S and SAI, as well, were significantly improved in both of the groups. SAS increment was significant only in the risperidone group (*P* < 0.02). *Conclusion*. While both of olanzapine and risperidone were equally effective for improvement of positive symptoms and insight, olanzapine showed superior efficacy with respect to negative symptoms, along with lesser extrapyramidal side effects, in comparison with risperidone.

## 1. Introduction

Schizophrenia is characterized by its chronic recurring course [1]. In addition, as many as 30–40% of such patients may exhibit an insufficient or poor response to conventional antipsychotics [2] and up to 50% of them may experience serious side effects by such treatments [3]. So the focus of new drug development for treatment of schizophrenia has shifted to synthesize compounds capable of alleviating negative symptoms, which are commonly unresponsive to classical antipsychotics, and to synthesize compounds less likely to produce extrapyramidal side effects. At the beginning, atypical antipsychotics seemed to be more efficient than conventional antipsychotics, but in a meta-analysis for comparing the effects of first-generation antipsychotics with second-generation ones, the latter cluster was found to be no more efficacious than the first-generation drugs in schizophrenic patients, even with respect to negative symptoms [4].

Olanzapine is a thienobenzodiazepine with a high affinity for serotonin 5-HT2, histamine H1, α1-adrenergic, D1, and D2 dopamine receptors [5]. Controlled clinical trials have shown that it has better efficacy and healthier side-effect profile than haloperidol and according to some studies seems to be more efficient in the management of negative symptoms [6–8]. Risperidone is a benzisoxazole derivative. Its greatest affinity is for serotonin 5-HT2, histamine H1, α1-adrenergic, and dopamine D2 sites. In some clinical studies, it has been shown to be superior to typical antipsychotics [9]. Both risperidone and olanzapine have been shown to be well tolerated and efficacious in the treatment of psychotic disorders [6, 7, 9, 10]. A variety of assessments for comparing these two atypical antipsychotics have resulted in diverse outcomes. For example, Tran et al. [10] and Gureje et al. [11] had found olanzapine to have a risk-versus-benefit advantage compared to risperidone. In this regard, subjects meeting diagnostic criteria for schizophrenia, schizoaffective or schizophreniform disorder were measured with the Positive and Negative Syndrome Scale (PANSS). According to Tran, the benefit of olanzapine was due to its greater efficacy, noticeable improvement of negative symptoms, higher response rate, better maintenance of treatment, and finally lower incidence of adverse effects like extrapyramidal side effects, hyperprolactinemia, and sexual dysfunction [10]. Also, Edgell et al. [12] and Rascati et al. [13] found that olanzapine-treated patients were more likely to sustain treatment versus risperidone-treated patients and Feldman et al. [14], as well, had found olanzapine to be more efficacious than risperidone in improvement of negative symptoms in older patients.

But conversely, in parallel comparisons, Taylor et al. [15], Kasper et al. [16], and Conley and Mahmoud [17] generally found equivalent clinical outcome for both of olanzapine- and risperidone-treated patients. So in the present assessment and based on the aforementioned controversies, the safety and efficacy of risperidone and olanzapine were compared once more in a sample of schizophrenic patients, looking for additional convincing proof regarding this important matter.

## 2. Method

This study was approved by University's Medical Ethics Committee. Sixty female in-patients, as accessible sample in the chronic ward of the hospital, after full explanation of the procedure for them and obtaining signed informed consent, and a minimum of 10–14-day washout period, entered randomly into one of the assigned groups, for random allocation to olanzapine or risperidone (*n* = 30 in each group). Patients were diagnosed as schizophrenic, according to* Diagnostic and Statistical Manual of Mental Disorders*, 4th edition, text revision criteria. Previous drugs of the patients consisted of a series of first-generation antipsychotics, including perphenazine, haloperidol, trifluoperazine, and chlorpromazine. The appraisal had been done through a double-blind, 12-week trial, while the patients, staff, and assessor were unaware of the prescribed drugs that were packed into identical capsules. No other psychotropic drug or psychosocial intervention, during the trial, was administrated for them. Scale for Assessment of Positive Symptoms (SAPS) and Scale for Assessment of Negative Symptoms (SANS) were used as the primary outcome measures [18]. Schedule for Assessment of Insight (SAI) [19], Clinical Global Impressions-Severity Scale (CGI-S) [20], and finally Simpson Angus Scale (SAS) [21] were also employed as secondary scales. The study duration was 12 weeks, and the patients were assessed by means of SAPS and SANS at baseline (week 0) and at weeks 4, 8, and 12. The other scales were scored at baseline and at the end of the assessment. Exclusion criteria included DSM-IV-TR axis I diagnosis other than schizophrenia, documented medical or neurological disease, utilization of atypical neuroleptics or concomitant therapy such as mood stabilizers or antidepressants, and finally any case with depot antipsychotics. Both of these drugs were prescribed according to practice guidelines and standard-titration protocols [22] and in accordance with the following regimen: 1 mg/day of risperidone or 5 mg/day of olanzapine at baseline up to 2 mg/day of risperidone and 10 mg/day olanzapine at the end of the first week. Weekly interval increments of 2 mg for risperidone and 5 mg for olanzapine, individually and according to clinical situation, were up to maximum of 8 mg and 25 mg for risperidone and olanzapine, respectively, at week 5. The 5th week dosage remained constant up to the end of the study.

## 3. Statistical Analysis

Patients were compared on baseline characteristics by means of *t*-tests. The primary analysis was carried out according to the mixed-effect model for repeated measure (MMRM), which estimates with comparatively small bias, in comparison with last observation carried forward (LOCF) approach, and controls type I error rates at a nominal level in the presence of missing completely at random (MCAR) or missing at random (MAR) and some possibility of missing not at random (MNAR) data. Treatment efficacy, as well, was analyzed by paired and nonpaired *t*-tests in intragroup and between-group comparison of means, respectively. Statistical significance was defined as a 2-sided *P* value < or = to 0.05. Cohen's standard (*d*) and correlation measures of effect size (*r*) were used for comparing baseline to end-point changes in primary outcome measures. MedCalc version 9.4.1.0 and OpenStat version 1.0.0.0 were used as statistical software tools for analysis.

## 4. Results

Analysis for efficacy was based on data from equal number of patients in olanzapine and risperidone groups. Groups were initially comparable and demographic and diagnostic variables were analogous (Table 1). Five patients (16%) in the olanzapine group and 6 patients (20%) in the risperidone group left the experiment in the second half of the trial due to unwillingness or adverse effect of the prescribed drugs.

Clinical improvement, defined as a 20% reduction in total scores of SAPS and SANS, was seen, respectively, in 86.66% and 56.66% of the cases in the olanzapine group and 73.33% and 36.66% of them in the risperidone group at the end of the assessment. Decrement of mean total score of SAPS was around %14.78 and %12.83 for olanzapine and risperidone, respectively. According to the findings, both of olanzapine and risperidone were significantly efficient in the improvement of positive symptoms (*P* < 0.0001). Decrement of mean total score of SANS as well was around %8.62 and %3.91 for olanzapine and risperidone, respectively. Analysis showed that negative symptoms in the present assessment improved significantly by olanzapine (*P* < 0.0003), while it was not so with respect to risperidone (*P* < 0.08) (Table 2).

Between-group analysis as well showed significant benefits of olanzapine versus risperidone at week 12 regarding SANS (*P* < 0.0052), while it was not so with respect to SAPS (*P* < 0.11) (Table 3).

CGI-S, as well, was significantly improved in both of the groups (*P* < 0.05 and *P* < 0.03 for olanzapine and risperidone, resp.) (Table 2). Similarly, SAI showed significant improvement by olanzapine (*P* < 0.003) and risperidone (*P* < 0.05) at week 12. Besides, SAS showed increase of extrapyramidal symptoms in both of the groups with 13.08% and 32.87% increment by olanzapine and risperidone, respectively, which was only significant in the risperidone group (*P* < 0.02) (Table 2). Between-group analysis, as well, showed significantly poorer state for risperidone vis-à-vis olanzapine (*P* < 0.04) (Table 3).

Since the sample size was small, the effect size (ES) was analyzed for changes on the primary outcome measures at the end of experiment. Results showed a large (*d* or *r* = or >0.8 or 0.3, resp.) readily observable improvement of SAPS and SANS by olanzapine and large and small (*d* or *r* = or <0.2 or 0.1, resp.) improvement of SAPS and SANS, respectively, by risperidone. The mean modal dose of olanzapine and risperidone during the present assessment was 20.49 ± 4.51 mg/day and 6.32 ± 1.68 mg/day, respectively. Throughout this study, extrapyramidal symptoms, mainly stiffness and tremor, were reported as an adverse event for 48.33% of the cases in the risperidone group and 16.66% of them in the olanzapine group, which was significantly more prevalent in the first group (*P* < 0.03). On the other hand, weight gain was significantly more evident in patients treated with olanzapine (46.66%) in comparison with risperidone (16.66%) (*P* < 0.03). Also, mean weight gain was significantly more in olanzapine group (3.4 +/− 0.8 kg) in comparison with risperidone group (0.7 +/− 0.2 kg) (*P* < 0.0001). Post hoc power analysis showed an intermediary power = 0.60 with respect to this trial.

## 5. Discussion

The primary objective of this study was to compare once more efficacy and safety of olanzapine and risperidone in schizophrenic patients, since there was no constant deduction so far, based on the results of the previous analogous studies. In essence and according to final outcomes, both of olanzapine and risperidone were significantly effective in reducing the severity of overall psychotic symptoms, while as regards improvement of negative symptoms and extrapyramidal side effects, olanzapine was shown to be more advantageous than risperidone. In this regard, maybe insignificant improvement of negative symptoms by risperidone was a bit due to intensification of secondary negative symptoms, which interfered with or concealed the improvement of primary ones. Anyway, these findings are somewhat comparable to another analogous double-blind, 28-week study on 339 patients who met DSM-IV criteria for schizophrenia, schizophreniform disorder, or schizoaffective disorder [10]. While that study reported a significantly greater proportion of olanzapine-treated patients achieving remarkable improvement in PANSS, better enhancement in SANS, and considerably a lower amount of EPS, in the present assessment there was no significant difference between olanzapine and risperidone with respect to improvement of positive symptoms or insight. There are additional studies as well with varying outcomes. For example, though in an 8-week head-to-head clinical trial comparing olanzapine with risperidone on 377 patients who met DSM-IV criteria for schizophrenia or schizoaffective disorder, there was no significant difference among them with respect to efficacy measures or extrapyramidal side effects [17], in the current appraisal significant worsening of such kind of adverse effects was evident only by risperidone. In the same way, another open-label, head-to-head study of olanzapine versus risperidone concluded that although there was greater improvement in psychotic symptoms in the risperidone group, there was significant increase in akathisia as well in the same group compared to olanzapine-treated cases [23]. Likewise, there are additional studies that have similar assumptions in support of risperidone [14, 15]. Maybe, applying different efficacy measures with various psychometric properties and different samples using unlike diagnostic criteria can explain to some extent these inconsistent results. In addition, since the weight gain was noticeably greater in the olanzapine group and this problem may well be an important trouble, perhaps, especially for women, such an adverse effect and related metabolic side effects, should always be taken into account by clinicians. The same vigilance also could be reasonable regarding extrapyramidal symptoms that were more challenging here with respect to risperidone. Hence, proper prescription of antipsychotics for special target group of patients cannot be independent of their potential side effect. While in comparison with the previous studies, the outcome of the present assessment may not be shown to be more decisive and free from controversy, it may propose the necessity of more parallel trials or meta-analytic studies regarding comparisons between atypical antipsychotics. Also, restriction of diagnosis to only schizophrenia, instead of whole of schizophrenia, schizoaffective and schizophreniform disorders, which was usual in the aforesaid trials, could endorse a more precise tryout in the present assessment. Besides, equal efficacy of these two second-generation antipsychotics regarding improvement of positive symptoms and insight may perhaps weaken any kind of irrational clinical preference regarding picking one of them as first choice, and, then again, significant improvement of negative symptoms by olanzapine may well show a better choice for schizophrenic patients with predominantly negative symptoms in longitudinal course. Also, strengthening of extrapyramidal side effects by risperidone may possibly classify it as antipsychotic that can increase the risk of tardive dyskinesia in susceptible patients. Small sample size, short duration of assessment, gender-based sampling, and lack of placebo arm, which may have significant impact on the assay sensitivity of the study and artificially inflate results in an active comparator trial, were among the weak points of this trial. Further analogous trials in future possibly will improve our knowledge in this regard.

## 6. Conclusion

While both of olanzapine and risperidone were equally effective for the treatment of patients with schizophrenia, olanzapine showed superior efficacy with respect to negative symptoms, along with lesser extrapyramidal side effects, in comparison with risperidone.

## Figures and Tables

**Table 1:** Demographic characteristics of patients participating in olanzapine and risperidone groups.

Variables	Olanzapine (*N* = 30)	Risperidone (*N* = 30)	*T*	*P*	95% CI
Age, y	36.89 ± 3.52	38.44 ± 4.91	1.283	0.205	−0.763, 3.481
Age at onset, y	22.48 ± 3.74	23.62 ± 4.91	0.923	0.360	−1.25, 3.17
Duration of illness, y	6.83 ± 1.63	6.39 ± 1.58	0.969	0.337	−2.28, 3.24
Number of prior episodes Mean ± SD	7.18 ± 2.13	6.82 ± 1.51	0.65	0.51	−1.46, 0.74
Baseline SAPS	63.61 ± 3.86	62.19 ± 4.11	1.379	0.1731	−0.64, 3.48
Baseline SANS	46.26 ± 3.37	46.83 ± 3.75	−0.619	0.5382	−2.41, 1.27
Baseline SAI	3.49 ± 0.61	3.51 ± 1.03	−0.092	0.9274	−0.46, 0.42
Baseline SAS	0.35 ± 0.76	0.35 ± 0.76	0.000	1.0000	−0.39, 0.39
Baseline CGI-S	3.65 ± 1.16	3.25 ± 0.12	1.879	0.0653	−0.03, 0.83

SAPS: Scale for Assessment of Positive Symptoms; SANS: Scale for Assessment of Negative Symptoms; CGI-S: Clinical Global Impressions-Severity Scale; SAI: Schedule for Assessment of Insight; and SAS: Simpson Angus Scale.

**Table 2:** Intragroup analysis of different outcome measures between baseline and week 12.

Measure	Olanzapine Baseline	Olanzapine Week 12	*T*	*P*	95% CI	Risperidone Baseline	Risperidone Week 12	*T*	*P*	95% CI
SAPS	63.61 ± 3.86	54.96 ± 3.42	9.187	0.0001	6.77, 10.53	62.19 ± 4.11	56.31 ± 3.02	6.315	0.0001	4.02, 7.74
SANS	46.26 ± 3.37	42.74 ± 3.69	3.858	0.0003	1.69, 5.35	46.83 ± 3.75	45.28 ± 3.06	1.754	0.08	−0.22, 3.32
CGI-S	3.65 ± 1.16	3.10 ± 1.03	1.942	0.05	−0.02, 1.12	3.25 ± 0.12	3.17 ± 0.16	2.191	0.03	0.01, 0.15
SAI	3.49 ± 0.61	3.83 ± 0.11	−3.004	0.003	−0.57, −0.11	3.51 ± 1.03	3.97 ± 0.74	−1.987	0.05	−0.92, 0.00
SAS	0.35 ± 0.76	0.38 ± 0.02	−0.216	0.82	−0.31, 0.25	0.36 ± 0.11	0.45 ± 0.19	−2.245	0.02	−0.17, −0.01

SAPS: Scale for Assessment of Positive Symptoms; SANS: Scale for Assessment of Negative Symptoms; CGI-S: Clinical Global Impressions-Severity Scale; SAI: Schedule for Assessment of Insight; and SAS: Simpson Angus Scale.

**Table 3:** Between-group analysis of different outcome measures at weeks 4, 8, and 12.

Measures	Olanzapine *N* = 30	Risperidone *N* = 30	*T*	*P*	95% CI
SAPS-4th week	61.44 ± 3.92	61.23 ± 3.61	0.216	0.82	−1.74, 2.16
SAPS-8th week	60.27 ± 3.82	60.98 ± 3.58	−0.743	0.46	−2.62, 1.20
SAPS-12th week	54.96 ± 3.42	56.31 ± 3.02	−1.621	0.11	−3.02, 0.32
SANS-4th week	45.19 ± 2.73	45.69 ± 2.29	−0.769	0.44	−1.80, 0.80
SANS-8th week	44.91 ± 3.37	45.76 ± 3.33	−0.983	0.32	−2.58, 0.88
SANS-12th week	42.74 ± 3.69	45.28 ± 3.06	−2.902	0.005	−4.29, −0.79
SAI-12th week	3.83 ± 0.11	3.97 ± 0.74	−1.025	0.30	−0.41, 0.13
SAS-12th week	0.38 ± 0.02	0.45 ± 0.19	−2.007	0.04	−0.14, −0.00
CGI-S-12th week	3.10 ± 1.03	3.17 ± 0.16	−0.368	0.71	−0.45, 0.31

SAPS: Scale for Assessment of Positive Symptoms; SANS: Scale for Assessment of Negative Symptoms; CGI-S: Clinical Global Impressions-Severity Scale; SAI: Schedule for Assessment of Insight; SAS: Simpson Angus Scale.
